# Insights into canine rabies vaccination Disparities in Sierra Leone: A cross-sectional household study

**DOI:** 10.1371/journal.pntd.0012332

**Published:** 2024-07-19

**Authors:** Philip P. Mshelbwala, Kinley Wangdi, Joseph A. Bunting-Graden, Saidu Bamayange, Andrew M. Adamu, Suman D. Gupta, Roland Suluku, Cornelius S. Adamu, J. Scott Weese, Charles E. Rupprecht, Nicholas J. Clark

**Affiliations:** 1 Faculty of Veterinary Medicine, University of Abuja, Abuja, Nigeria; 2 NSW Department of Primary Industries, Orange, Australia; 3 School of Veterinary Science, The University of Queensland, Gatton, Australia; 4 HEAL Global Research Centre, Health Research Institute, Faculty of Health, University of Canberra, Bruce, Australia; 5 Directorate of Health Security and Emergencies, Ministry of Health and Sanitation, Freetown, Sierra Leone; 6 Livestock & Veterinary Services Division Ministry of Agriculture & Food Security, Freetown, Sierra Leone; 7 Australian Institute of Tropical Health and Medicine, James Cook University, Townsville, Australia; 8 College of Public Health Medical and Veterinary Sciences, James Cook University, Townsville, Australia; 9 School of Agricultural, Environmental and Veterinary Sciences, Faculty of Science and Health, Charles Sturt University, Wagga Wagga, NSW, Australia; 10 Gulbali Institute, Charles Sturt University, Wagga Wagga, NSW, Australia; 11 Njala University, Njala, Sierra Leone; 12 Department of Pathobiology, Ontario Veterinary College, Guelph, Canada; 13 College of Forestry, Wildlife & Environment, College of Veterinary Medicine, Auburn University, Auburn, Alabama, United States of America; US Department of Agriculture, UNITED STATES OF AMERICA

## Abstract

Annually, Sierra Leone records an estimated 301 human fatalities due to rabies. Canine vaccination is crucial for rabies prevention and control efforts. However, considerable variability exists in vaccination rates. Reasons for this variation remain unclear. We conducted a cross-sectional study across 2,558 dog-owning households (HHs) to provide insights into factors influencing canine vaccination for targeted prevention and control towards elimination by 2030. First, we described dog ownership practices, then built a probabilistic model to understand factors associated with dog vaccination, and finally used a spatial scan statistic to identify spatial clusters where vaccination rates were low. Our results indicated that only 14% (358/2,558) of participating HHs had fully vaccinated their dogs against rabies. The probability of dog vaccination increased when comparing civil servants to private workers/artisans, with an Odds Ratio(OR) of 1.14 (95% credible interval (Crl) of 0.82–1.56), residing in locations with a veterinary establishment vs. none (OR = 6.43, 95% Crl (4.97–8.35), providing care to dogs vs. allowing dogs to roam freely (OR = 2.38, 95% Crl(1.80–3.17) and owning a single dog vs multiple dogs (OR = 1.20, 95 Crl (0.92–1.56). Conversely, there was a decrease in the estimated probability of vaccination when comparing dog owners located in rural vs. urban areas (OR = 0.58, CrI 95% (0.43–0.78). Latent understanding, a measure of overall understanding of rabies virus, which we estimated using participant education levels and responses to questions about rabies epidemiology, was also an important predictor of vaccination probability (OR = 1.44, 95% Crl (1.04–2.07). The spatial analysis identified high-risk clusters for low vaccination in the cities of Moyamba, with a radius of 40 km, a relative risk (RR) of 1.10, and Bo, with a radius of 19.9 km with RR of 1.11. These data do not support Sierra Leone reaching the 2030 goal of human rabies elimination caused by dogs. Our study highlights a critical need for public outreach and education, improved vaccination rates, increased accessibility to veterinary services, and targeted interventions in Bo and Moyamba to support rabies prevention and control efforts.

## Author summary

Canine vaccination plays a vital role in rabies prevention and control efforts; however, significant variability exists in vaccination rates across Sierra Leone. To address this issue, we conducted a cross-sectional study involving 2,558 dog-owning households to understand the factors influencing canine vaccination and identify areas with low vaccination rates. Our findings revealed that vaccination probability increased among civil servants compared to private workers/artisans, those residing in areas with veterinary establishments, those providing care to dogs, and those owning a single dog. Conversely, vaccination probability decreased among dog owners in rural areas compared to urban areas. Latent understanding, a measure of overall understanding of rabies virus, which we estimated using participant education levels and responses to questions about rabies epidemiology, was also an important predictor of vaccination probability. Spatial analysis identified high-risk clusters for low vaccination rates in Moyamba and Bo. These findings underscore the urgent need for public outreach, education initiatives, improved vaccination rates, enhanced veterinary accessibility, and targeted interventions in Bo and Moyamba to combat rabies effectively.

## Introduction

Annually, tens of thousands of people in low- and middle-income countries (LMICs) face the grim threat of rabies-related deaths [1,2]. Most fatalities stem from bites by rabid dogs [2]. This zoonosis not only exacts a heavy toll on human lives but also imposes a substantial economic burden, with an estimated 3.7 million disability-adjusted life years lost and 8.6 billion US dollars in annual economic losses [2]. Dog vaccination is the most effective control measure in mitigating the spread of rabies [3,4]. While canine-mediated rabies has been successfully eliminated in all developed nations, many LMICs, particularly across sub-Saharan Africa, continue to grapple with this challenge [5]. In 2015, the Pan-African Rabies Control Network (PARACON) was established under the Global Alliance for Rabies Control (GARC) secretariat to unite groups within the region, enhance scientific expertise, and develop strategies to eliminate human deaths from canine rabies [6]. The PARACON was in line with the Global Strategic Plan to end human deaths from dog-mediated rabies by 2030 (*Zero by 30*, *ZBT*) and United Nations Sustainable Development Goals (UN SDGs). Specifically, SDG No. 3 encompasses plans to "ensure healthy lives and promote well-being for all at all ages," with a specific contribution to target 3.3, to: "…end the epidemics of AIDS, tuberculosis, malaria, and neglected tropical diseases, and combat hepatitis, water-borne diseases, and other communicable diseases by 2030”". [7,8]. United Against Rabies (UAR) was formed most recently to support countries’ progress towards *ZBT* [9]. However, evidence suggests that rabies remains uncontrolled throughout the region, with only a handful of countries, such as Namibia, progressing towards elimination [5,10–12].

In Sierra Leone, the first reported rabies case dates to 1949, marking the initiation of a persistent challenge that has grown in magnitude [13]. The first vaccination campaign took place in 1954 in the capital of Freetown, signifying the early acknowledgment of the issue [13]. However, subsequent efforts have been characterised by erratic reactive dog vaccination efforts, often triggered by sporadic outbreaks centred around World Rabies Day (WRD) [13]. Over time, human and animal rabies cases have increased, particularly across major towns, attributed to the prevalence of free-roaming dogs, increased population density, low vaccination rates, and a less-than-ideal level of community knowledge [13].

A study conducted in 2015 estimated an alarming 301 annual rabies deaths in Sierra Leone, emphasising the severity of the issue [2]. Dog vaccination coverage was reported to be a mere 0.8%, indicating a critical gap in preventive measures. Hospital dog bite cases between 1995 and 2001 for two regional hospitals in Freetown and Bo indicated a steady increase in confirmed human rabies, reaching a peak in 2001 [14]. Males and children between 0 and 15 years were identified as the most significant risk group for dog bites during this period [14].

A random survey conducted between December 2001 and May 2002 involving 315 households (HHs) in Freetown revealed a stark lack of awareness and preventive measures [14]. None of the HHs had vaccinated their dogs against rabies or were aware of the importance of regular vaccination [14].

Achieving necessary herd immunity, with at least 70% of dogs vaccinated annually via mass dog vaccination, is critical to breaking the transmission of dog-mediated human rabies [15]. To inform effective planning and implementation of mass vaccination campaigns, it is crucial to understand dog population characteristics, including factors associated with vaccination decisions and locations with low vaccination rates [16,17]. Sierra Leone has recently developed its national strategic plan for rabies prevention and control. However, the absence of evidence regarding dog characteristics, including access to vaccine, factors influencing vaccination, and locations with low vaccination rates, has hindered implementation efforts, particularly in the context of limited available resources. This study aimed to describe dog ownership practices, model the factors associated with canine vaccination, and identify clusters of low vaccination rates to inform targeted mass vaccination campaigns. Our objective was that the study findings would guide the planning of targeted mass dog vaccination campaigns to enhance canine vaccination rates in Sierra Leone in line with *ZBT*.

## Materials and methods

### Ethical considerations

Research permission and ethical clearance were obtained from the Directorate of Livestock and Veterinary Services within the Ministry of Agriculture & Food Security, Sierra Leone, and the University of Abuja Ethics Committee on Animal Use (UAECAU/2023/004), Nigeria. Additionally, we obtained verbal consent from all survey participants.

### Study location

Sierra Leone is a West African country along the Atlantic Ocean, between latitudes 6° 55’ and 10° 00’ N and longitude 10° 16’ and 13° 18’ W, covering a geographic area of ~72,000 km^2^. The country is bounded by the Republic of Guinea to the north and northeast, Liberia to the southeast, and the Atlantic Ocean to the south and southwest (running from the north right down to the south on the west of Sierra Leone). The country is divided into five provinces and 16 districts. Sierra Leone has an estimated human population of 8,141,343 as of July 2021, with an average growth rate of 2.0% between 1990 and 2010.

### Sampling and data collection

We conducted a cross-sectional survey from July 2022 to December 2023 across Eastern, Southern, Northern-Western, and Western area provinces (S1 Fig). Rural and urban settlements were selected based on reports of dog bites and cat bites [13]. We used multi-stage sampling on each selected street, commencing at the initial residences on both sides. Our approach involved systematically selecting every tenth household for interviews with adult residents, following the methodology described by Okoh (1986) [18]. However, in cases where a systematic approach was impractical due to the absence of clearly defined road networks, we relied on informal directions provided by community members to identify HHs with dogs [17]. If the tenth household did not have dogs or residents declined to participate in the interview, we moved on to the next house in sequence. Before the survey, the questionnaire was pretested among 41 dog owners in Njala (Southern Province) to test for consistency and validity. However, we relied on community members for direction, given that only a few areas of Freetown had well-defined streets. The questionnaire was developed in English in collaboration with experts within and outside Sierra Leone, who administered the survey in English, Krio, Mende, Temne or Limba. The questionnaire was administered on electronic handheld devices (Android phones) using the Kobo toolbox.

Interviews were conducted with adult household members proficient in English, Krio, Mende, Temne or Limba. If the participant had difficulty understanding any questions, they were translated into the language of the respondent’s choice. The head of each of the selected HHs was interviewed. In their absence, another adult household member willing to participate in the study was interviewed in accordance with the Helsinki Declaration (2001) [19]. Thirteen Njala University students from each province were selected and trained as enumerators based on their ability to speak English, the official language of Sierra Leone, and any of the commonly spoken Krio or Theme languages. Survey data were uploaded into a real-time data management system using mobile data. The survey targeted three main categories of information. Firstly, we collected sociodemographic details of the dog owner, including age, gender, education level (categorical), occupation (categorical), including students, civil servants, private workers and artisans (for analysis purposes, we combined Private/Artisans) and the number of people (discrete) and dogs residing in the household (discrete). Secondly, the questionnaire pertained to dog-keeping practices, including the primary purpose of the dog (categorical) and its confinement status (categorical)—where confined dogs were securely enclosed, partially confined dogs had limited boundaries, and unconfined dogs had unrestricted movement. For analysis purposes, we reclassified them into two: confined (partially and fully confined dogs) and unconfined. Access to veterinary care (Is there a veterinary establishment in your location? ‘Yes’, ‘no’, and ‘I am not aware’) (categorical) was reclassified to a binary predictor by combining ‘not aware’ and ‘no’ vs ‘yes’ for analysis. Data collected also included the dog’s vaccination status (binary), history of dog bite to the respondent or respondent’s household members (binary), and the preferred treatment option in case of a dog bite (binary). Lastly, a series of questions targeted respondents’ knowledge about rabies, covering familiarity with the term ’rabies’ (binary), the mode of rabies virus (RABV) transmission (binary), the types of hosts susceptible to RABV (ordinal), previous exposure to a dog bite (binary), and post-exposure care following a dog bite. Respondents who indicated that their dogs were not vaccinated against rabies were asked follow-up questions about the reasons, including the vaccine cost, lack of knowledge on accessing the vaccine, and a perception that vaccination was important and significant. Details of the questionnaire used for this survey can be accessed here. To account for the impact of dog owners’ spatial distribution on their choices regarding canine vaccination, we obtained the urban extent grid raster map at 0.00833 degrees (30 arc seconds) resolution, delineating urban and rural locations in Sierra Leone from the Global Rural-Urban Mapping Project [20]. All the surveyed HHs were geo-coded and superimposed over the urban map to classify the location of the HHs (urban vs rural) using the SF package R version 4.1.2 [21].

### Causal framework

**Fig 1:**
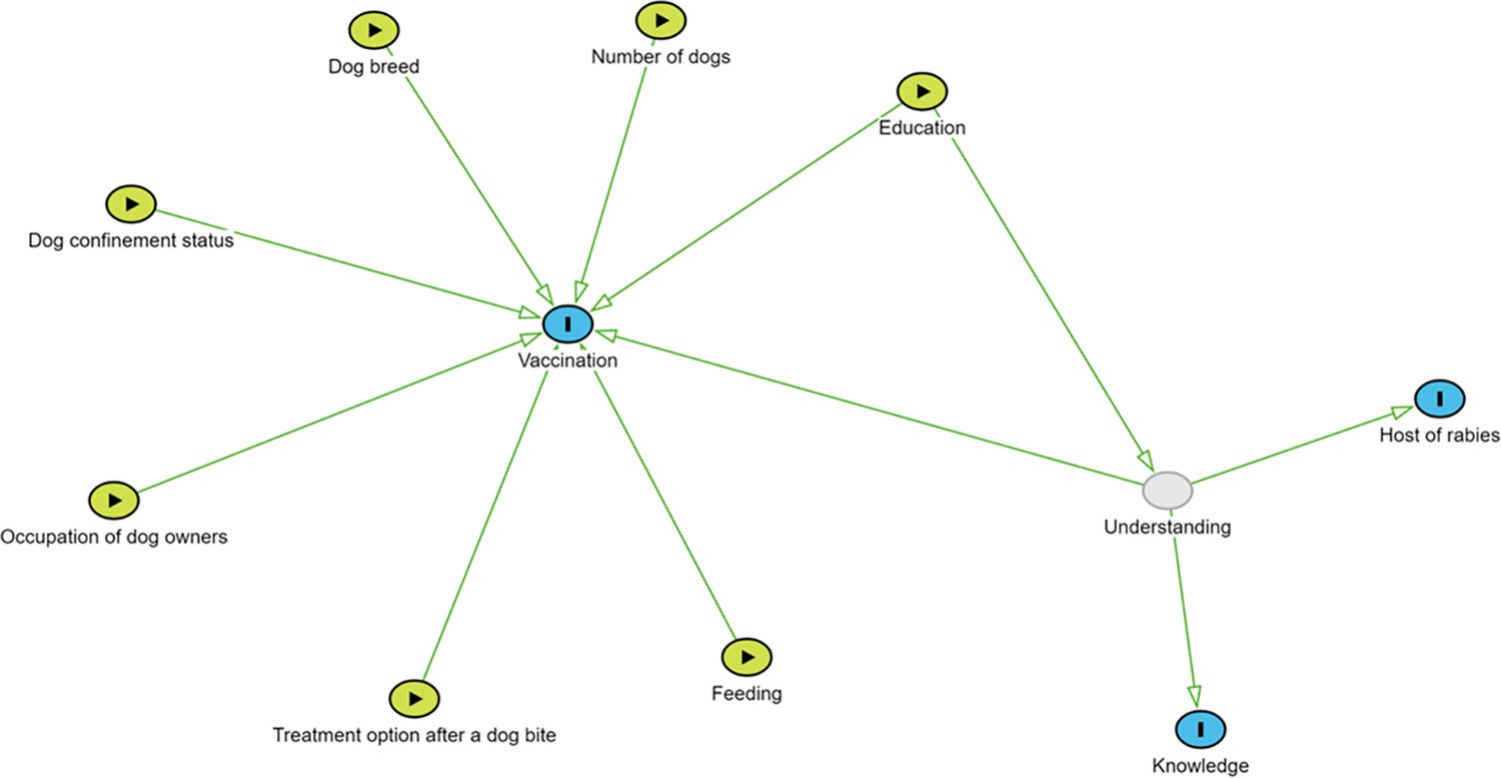
Directed Acyclic Graph (DAG) illustrates how rabies vaccination status is hypothesised to vary with an observed confounder education, primary use of the dog, number of dogs in the house, dog’s confinement status, feeding and caring methods (care), occupation of the dog owner and treatment option following a dog bite, rural or urban residence, and a latent understanding(understanding)—representing the respondents’ general comprehension of rabies. The blue circles represent outcome variables, the green circles represent exposures (i.e., predictor variables), and the grey circles represent unobserved variables. Generally, green lines represent the front door path or the causal effect of interest.

We used DAGitty v3 to construct a causal diagram to guide data collection and analysis. Our Directed Acyclic Graph (DAG) delineated hypothesised relationships between canine rabies vaccination and dog ownership factors associated with vaccination and non-vaccination. The DAG is a graphical exploratory generative model that permits the derivation of a causal estimand [22,23]. For this study, we used DAG over stepwise selection processes because DAG provides a rigorous framework for representing and analysing causal relationships among variables, thereby reducing the risk of spurious correlations and selection bias inherent in stepwise methods [24,25]. Our DAG delineated hypothesised relationships between canine rabies vaccination and dog ownership factors associated with vaccination and non-vaccination. These factors included the number of dogs in a household, level of care, confinement status, owner’s education level, occupation, urban or rural residence, and health-seeking behaviour following a dog bite. Each of these factors was hypothesised to influence dog vaccination directly. We also hypothesised that each owner’s knowledge about RABV may act as an additional predictor of their choice to vaccinate or not. As we could not directly measure this knowledge, we modelled it as a latent variable that was informed by three observed responses: awareness of the term rabies; mode of RABV transmission through dog bites, and ordinal responses regarding the host of rabies. The ordinal responses included the perception that rabies affects both humans and animals (highest score), rabies affects only humans or only animals (medium score) and being unaware of the host of rabies (lowest score). We constructed binary and ordinal logistic regressions to model these components, contributing to the unobserved latent understanding, which directly influences dog vaccination practices, with education as a confounder, affecting both latent understanding and vaccination outcome (Fig 1).

### Statistical analysis

Bayesian inference enables the estimation of a joint posterior distribution over unknown parameters in a statistical model, which is the product of the prior distribution and the likelihood of the data. We constructed a joint probabilistic model using Stan version 2.26.1 in the R Studio environment (The R Foundation for Statistical Computing) [26,27]. We used Stan because of its flexibility in specifying complex statistical models, efficiency in sampling from posterior distributions, scalability for handling large datasets, robust model diagnostics, and the support of an active open-source community [26]. We aimed to understand factors associated with dog vaccination, including latent understanding, a measure of dog owner’s unobserved overall understanding of rabies, which took several possible response distributions from our observed data, i.e., ordinal, categorical, binary and several dog ownership practices, that we modelled from our observed data as follows. First, we modelled the observed vector of binary responses to familiarity with rabies (*Familiarity*) as outcome variables assumed to be drawn from Bernoulli distributions with unknown parameters (*p*_*i*_), probability of being familiar with rabies. We modelled the *p*_*i*_s as directly depending on the latent understanding variable (Understanding) and intercept terms (α_*i*_) to capture systematic variation in average responses to each familiarity question:

         *For i in 1,2 … I familiarity with rabies questions*

               *Familiarity*_*i*_ ~ Bernoulli(*p*_familiarity[i]_)

             logit(*p*_familiarity[i]_) = α_familiarity[i]_ + *understanding*

     α_familiarity_ ~ Normal (-0.25, 1)

Second, it was important to improve inferences about the latent understanding variable. Our outcome of interest was an ordered response to a survey question about what kinds of host species are susceptible to RABV (host). Respondents who answered correctly (i.e., those who indicated that rabies affects both humans and animals) scored 3, those with one correct host scored 2, and those who answered ’neither human nor animal’ scored 1. We used an ordinal regression model that depended on latent knowledge (understanding) as a predictor. Ordinal outcomes can be modelled by introducing a latent continuous variable that is related to the observed outcomes via a Categorical observation model with a cumulative logit link function, where a set of internal cutpoints (*c*_*k*_) partitions the log-cumulative-odds of the *K* ordered response categories. We used a principled Dirichlet prior model on internal cutpoints, ensuring a robust prior model when ordinal data are weakly informative in some or all possible response categories [28].

         *For k in 1,2 … K ordered rabies host response categories*

               *host* ~ Categorical(*p*_host_)

                   *p*_host[__1__]_ = *q*_1_

             *p*_host[*k*]_ = *q*_*k*_*−q*_*k*-1_ for *K* > *k* > 1

             logit(*q*_*k*_) = *c*_*k*_−*understanding*

                  *c*_*k*_ ~ Dirichlet(*K*)

The latent *Understanding* variable was assumed to follow a normal distribution in which the mean was modelled using a linear predictor to capture our proposed dependency on education. The variance parameter (*σ*_*understanding*_) was not identifiable at values approaching 0 (which would result in the latent understanding variable perfectly matching the measured education variable), so we specified an informative uniform prior density to capture our belief that a person’s knowledge of rabies was strongly, but not solely, correlated with education:

         *understanding* ~ Normal (*μ*_*understanding*_, *σ*_*understanding*_)

               *μ*_*understanding*_ = β_*understanding*_ · *education*

                     *σ*_*understanding*_ ~ Uniform (0.5, 1)

                     β_*understanding*_ ~ Normal (0.25, 1)

The final component was also built with the assumption that the observed vector of outcome dog vaccination among dog owning HHs in Sierra Leone was drawn from a Bernoulli distribution with unknown parameters ’p,’ the probability of dog vaccination and depended on linear, additive effects of latent knowledge (understanding), confound (education), occupation of the dog owner, dog use, number of dogs, level of care provided to a dog, dog confinement status, the location of the dog owner (urban vs. rural), and the owner’s care-seeking practice following exposure to dog bite (allopathic vs. traditional). The intercept parameter (α_vaccinate_ ~ Normal (-0.75, 1) for our final model was assumed to follow a normal distribution with a mean of -0.75 and a standard deviation of 1, while the coefficient parameter (β_vaccinate_ ~ Normal (0.25, 1)) was assumed to follow a normal distribution with a mean of 0.25 and a standard deviation of 1. These prior distributions reflected our beliefs about these parameters before observing our data.

Our unit of analysis was individual HHs in Sierra Leone. In instances where HHs owned more than one dog, we categorised them as fully vaccinated only when at least 70% of their dogs had received rabies vaccination substantiated by valid vaccination certificates. This approach aligned with the World Health Organization’s recommendation of achieving at least 70% vaccination to interrupt canine RABV transmission [15].

To ensure our prior choices excluded unrealistic behaviours, we used previous reports on rabies that explored knowledge, attitudes, practices, and dog vaccination to simulate the expected data-generating process (S1 File).

### Spatial scan analysis

To identify locations of low vaccination rates, we used Kulldorff’s spatial scan statistic using SaTScan version 9. 6 [29]. The SaTScan uses moving circular or elliptical scanning windows of varying sizes to evaluate whether the observed frequency of positive cases within a window exceeded chance expectations [30]. This method allowed the identification of statistically significant clusters in spatial or spatiotemporal data. Our spatial analysis aimed to delineate clusters of HHs with low canine rabies vaccination to inform the planning of mass vaccination campaigns. The input dataset included the administrative shapefile of Sierra Leone, longitude and latitude coordinates of HHs with dogs, vaccination status, case (unvaccinated), control (vaccinated) in the World Geodetic Coordinate System of 1984, and the dog population in each HH. We used a Bernoulli spatial scan statistic, configuring the analysis with clusters of maximum size equivalent to 50% of the unvaccinated dog population. We incorporated 999 Monte Carlo replications for statistical significance testing to ensure a robust cluster definition, preventing geographical overlap in the identified clusters. The analysis assessed observed and expected positive cases inside and outside the scanning windows to detect clusters and estimate relative risk (RR). The SaTScan algorithm determined the most probable cluster for each household by selecting the window size with the highest Log-Likelihood Ratio (LLR), indicating spatial non-randomness. The SaTScan output for statistically significant clusters provided details including the location of the scanning window’s center, its radius, observed and expected positives within the circle, relative risk, LLR, and p-value. Statistically significant clusters were defined as those with a p-value less than 0.05. Subsequently, we employed QGIS (the Open Source Geospatial Foundation (OSGeo) to map the identified significant clusters.

## Results

### Demographic characteristics of survey participants and dog ownership practices

In the survey, 2,558 dog-owning HHs were interviewed. Table 1 contains information on the characteristics of the participants and their dogs and knowledge questions regarding rabies. The total number of people in the HHs was 24,166, while the total number of dogs was 8,791, resulting in a dog-to-human ratio of 1:2.7, approximately 1:3. On average, there were 3 dogs per HH. Most dog owners were male (59%, 1,491/2,558). About 49% were above 31 years of age (49%, 1,265/2,558). Only 32% (826/2,558) of the respondents reported having no formal education. Concerning rabies vaccination, only 14% (339/2,558) of the dog owning HHs had their dogs fully vaccinated. The major reason for non-vaccination among the 2,219 respondents whose dogs were not vaccinated was not knowing how to vaccinate their dogs (70%, 1,574/2,219). Other reasons included not considering rabies vaccination as important (18%, 388/2,219) and the cost of the vaccine (11%, 238/2219). Most (58%, 1,503/2,558) dog owners reported not having a livestock officer or veterinary establishment nearby.

**Table 1:** Demographic characteristics and dog ownership practices, including gender of dog owner, age, occupation education levels, number of dogs in each household(1 or >1), confinement status, use of dogs, and knowledge about rabies and veterinary care.

Characteristic		Vaccinated (%) *	Unvaccinated (%)	Total (%)
**Gender**				
	Female	150 (44.3)	917(41.3)	1,067(41.7)
	Male	189 (55.7)	1,302 (58.7)	1,491(58.3)
**Age of the owner (years)**				
	0–15	49 (14.5)	161(7.3)	210 (8.2)
	16–31	130 (38.3)	953(42.9)	1,083 (42.3)
	>31	160(47.2)	1,105(49.8)	1,265(49.5)
**Occupation**				
	Students	103(30.4)	294(13.2)	397(15.5)
	Civil servant	59(17.4)	444(20.0)	503(19.7)
	Private	78(23.0)	743(33.5)	821(32.1)
	Artisan	99(29.2)	738(33.3)	837(32.7)
**Level of education**				
	None	55(16.2)	771(34.7)	826(32.3)
	Primary	58(17.1)	334(15.1)	392(15.3)
	Secondary	100(29.5)	727(32.8)	827(32.3)
	Tertiary	126(37.2)	387(17.4)	513(20.1)
**Number of dogs in the household**				
	Only one dog	206(60.8)	1,065(48.0)	1,279 (50.0)
	More than one dog	133(39.2)	1,154(52.0)	1,279 (50.0)
**Is your dog under confinement?**				
	Partial confinement	116(34.2)	532(24.0)	648 (25.3)
	No	94(27.7)	1273(57.4)	1,367 (53.4)
	Yes	129(38.1)	414(18.6)	543 (21.2)
**Use of the dog?**				
	Breeding	4(1.2)	99(4.5)	103 (4.0)
	Security	261(77.0)	1414(63.7)	1675(65.5)
	Hunting	16(4.7)	386(17.4)	402(15.7)
	Pet	58(17.1)	320(14.4)	378(14.8)
**Have you heard about rabies?**				
	No	254(74.9)	1273(57.4)	1,527(59.7)
	Yes	85(25.1)	946(42.6)	1031(40.3)
**Rabies can infect the following?**				
	Only human	1(0.3)	67(3.0)	68(2.7)
	Only animals	27(8.0)	169(7.6)	196(7.6)
	Humans and animals	275(81.1)	1650(74.4)	1,925(75.3)
	Don’t know	36(10.6)	333(15.0)	369(14.4)
**Bites from an infected animal cannot spread rabies to other animals?**				
	True	76(22.4)	404(18.2)	480(18.8)
	False	263(77.6)	1815(81.8)	2,078(81.2)
**How do you feed the dog?**				
	Family left over	233(68.7)	1721(77.6)	1,954(76.4)
	Cook special pot	80(23.6)	262(11.8)	342(13.4)
	Sources food on its own	26(7.7)	236(10.6)	262(10.2)
**Is there a livestock officer or veterinary establishment at your location?**				
	Don’t know	39(11.5)	512(23.1)	551(21.5)
	No	135(39.8)	1,368(61.6)	1,503(58.8)
	Yes	165(48.7)	339(15.3)	504(19.7)
**If you or any member of your family were ever bitten by a dog which method of treatment would you prefer?**				
	Traditional treatment	87(25.7)	695(31.3)	782(30.6)
	Contemporary medicine	252(74.3)	1,524(68.7)	1,776(69.4)
**Province of the dog owner**				
	Eastern	172(14.7)	995(85.3)	1,167(45.6)
	Southern	165(14)	1015(86)	1,180 (46.1)
	Northwestern	11(12)	81(88)	92(3.6)
	Western area	10 (8.4)	109(91.6)	119(4.7)
**Location of the dog owner**				
	Rural	240(11.6)	1,836(88.4)	2,076(81.2)
	Urban	118(24.5)	364(75.5)	482 (18.8)

*vaccinated—70% of dogs vaccinated.

The survey findings indicated that awareness about rabies was limited, with only 40% (1,031/2,558) of the respondents having heard of the disease. When asked about the animals that could be infected by RABV, 75.3% (1,915) correctly identified humans and animals, while 14.4% (369/2,558) admitted not knowing, 2.3% (58/2,558) believed only humans could be infected, and 8% (196/2,558) believed only animals could be infected.

A large proportion of the respondents (81%, 2,078/2,558) believed that RABV cannot be transmitted to other animals through bites from a rabid animal. Additionally, 31% (780/2,558) preferred consulting traditional treatment over contemporary medicine in the event of someone getting bitten by a dog. Four percent (n = 124) of the respondents disclosed that someone in their household had experienced a dog bite in the last 2 years. Among the reported dog bites, 39.5% (49/124) involved household dogs, 37.9% (47/124) were attributed to neighbours’ dogs, and 22.6% (28/124) were associated with free-roaming dogs. Regarding interventions, post-exposure prophylaxis (PEP) was administered to most dog bite victims (72%, 89/124). Unfortunately, 50 respondents (who had prior knowledge of someone bitten by a rabid animal) reported knowing someone who had died from rabies. Of the dogs involved, 18 died, 21 were killed by a mob, 47 ran away, and an additional 38 could not be accounted for by respondents.

Among the owners of the 2,219 unvaccinated dogs, (29%, 743/2,219) were private workers and artisans. Dog owners with more than one dog had the highest proportion of unvaccinated dogs (52%, 1,154/2,219). Free-roaming dogs included a significant proportion of unvaccinated dogs (57%, 1,275/2,219). Most 64% (1,414/2,219) unvaccinated dogs were used for security purposes. Regions lacking veterinary establishments had a higher prevalence of unvaccinated dogs (62%, 1,368/2,219), and family members unbitten by dogs in the last two years indicated a substantial unvaccinated rate (81%, 1,805/2,219).

### Posterior distribution of factors associated with dog vaccination and the effect of latent understanding on education

**Fig 2:**
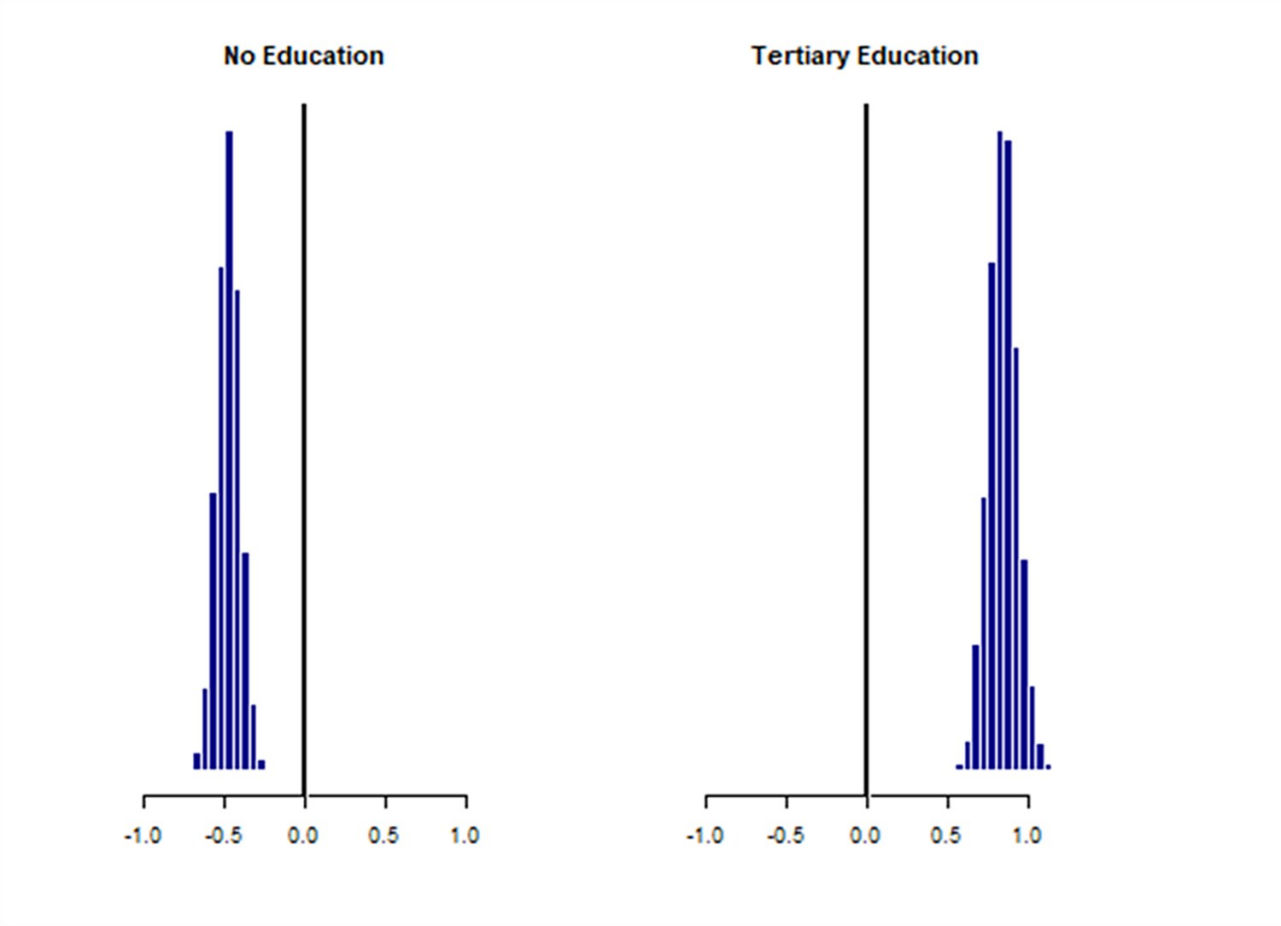
The posterior distribution of the effect of education on latent understanding of rabies demonstrated a positive association between latent understanding and tertiary education. No education was negatively associated with latent understanding.

Posterior distributions of estimated effects on the logit scale indicated that the probability of dog vaccination was predicted to be positively associated with employment as a civil servant compared to private worker/artisans, with an (OR = 1.14, 95% Crl of 0.82–1.56), preference for contemporary treatment in the event of a dog bite compared to traditional treatment (OR = 1.77, 95% Crl (1.27–2.47), ownership of a single dog compared to multiple dogs (OR = 1.20, 95% Crl (0.92–1.56), providing care to dog by way of providing meals compared to allowing dogs to roam freely (OR = 1.71, 95% Crl 1.24–2.38), dogs under full and partial confinement compared to those allowed to roam freely (OR = 2.38, 95%, Crl(1.80–3.17) and residing in areas with a veterinary establishment (OR = 6.43, 95% Crl (4.97–8.35)]. Residence in a rural location was negatively associated with dog vaccination (OR = 0.58, CrI (0.43–0.78). Also, latent understanding was positively associated with dog vaccination practices (OR = 1.44, 95% Crl (1.04–2.07)) (Table 2). Latent understanding was positively associated with tertiary education and negatively associated with no-education (Fig 2).

**Table 2:** Posterior distribution of estimated effects on the logit (probability of vaccination) among dog owners in Sierra Leone in 2023.

Characteristic	OR	2.5% Crl	97.5% Crl	Rhat
**What is your occupation?**				
Private/Artisans	**Reference**			
Students	1.14	0.82	1.56	1
Civil Servant	1.48	0.90	2.24	1
**What is your education level?**				
Secondary/Primary	**Reference**			
No education	1.44	0.98	2.13	1
Tertiary	1.45	0.95	2.17	1
**What treatment option would you opt for in the event of a dog bite?**				
Traditional treatment	**Reference**			
Allopathic medicine	1.77	1.27	2.47	1
**Number of dogs in the household**				
>1 dog	**Reference**			
One dog	1.20	0.92	1.56	1
**How do you feed your dog?**				
Leftover	**Reference**			
Cook special meal	1.71	1.24	2.38	1
Allow to roam	0.67	0.34	1.25	1
**What is the primary use of the dog?**				
Breeding and hunting	**Reference**			
Pet	0.89	0.52	1.57	1
Security	1.09	0.69	1.76	1
**Is your dog under confinement?**				
**No**	**Reference**			
Yes / Partial	2.38	1.80	3.17	1
**Awareness**				
Latent awareness	1.44	1.04	2.07	1
**Is there a veterinary establishment in your location?**				
No	**Reference**			
Yes	6.43	4.97	8.35	1
**Location of the HH**				
**Urban**	**Reference**			
Rural	0.58	0.43	0.78	1

Crl, (credible Interval), OR = odds ratio

### Cluster analysis

**Fig 3:**
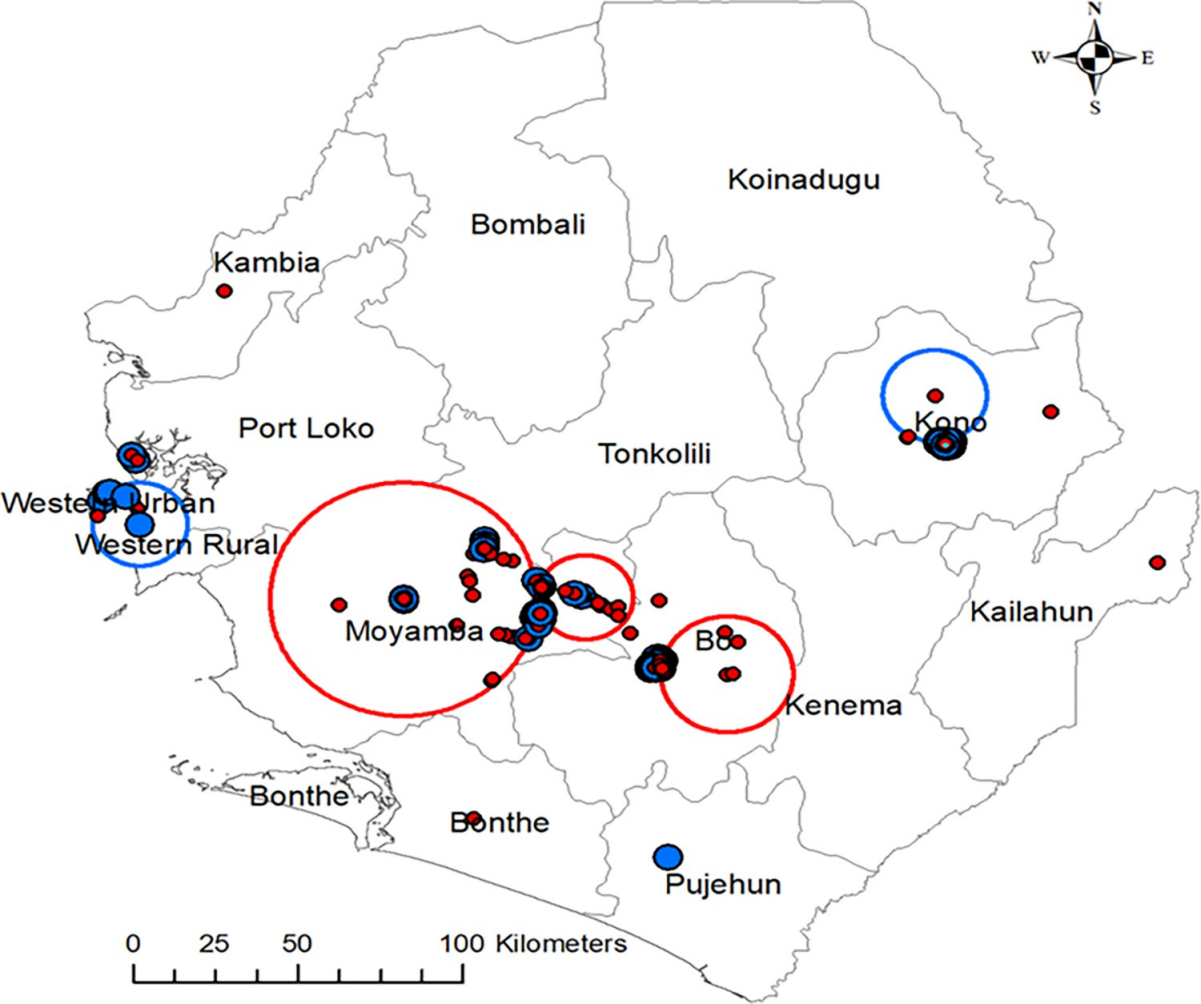
Spatial clusters of non-vaccination among dogs against rabies in Sierra Leone during 2023 revealed more likely clusters (depicted in red) in Moyamba with a radius of 40 km. Included are 626 dogs with a relative risk (RR) of 1.10. Bo (Southern Province) had a radius of 19.9 km and 206 dogs with an RR of 1.11. Areas such as the Kono District (Eastern Province), included 68 dogs, with RR of 0.68 and Freetown (Western Area), radius 20 km, with 68 dogs, with RR of 0.68 lower risk of non-vaccination. Map of Sierra Leone. The map was created using QGIS (the Open Source Geospatial Foundation (OSGeo). The shapefile was retrieved from DIVA-GIS (https://www.diva-gis.org/).

Spatial clusters of low vaccination among dogs against rabies revealed more likely clusters (depicted in red) in Moyamba with a radius of 40 km and 626 dogs and a RR of 1.10. We also observed low vaccination clusters in Bo (Southern Province), with a radius of 19.9 km and 206 dogs with an RR of 1.11. Low vaccination was also seen in the Kono District (Eastern Province), with 68 dogs and RR 0.68, and in Freetown (Western Area), with a radius of 20 km, 68 dogs, and RR of 0.68 (Blue circles, Fig 3).

## Discussion

Our research provided crucial evidence regarding the determinants of dog vaccination in Sierra Leone, offering essential insights for successfully implementing rabies control measures in the nation. This is the first effort to survey substantial HHs with canine ownership and build models to understand the dynamics of dog vaccination within the Sierra Leone context. Factors associated with canine vaccination were working as civil servants, having a latent understanding of rabies, residing in urban areas, providing care for dogs, and residing in an area with a veterinary establishment. In contrast, factors such as owning more than two dogs and a preference for traditional treatment following a dog bite were associated with non-vaccination. We also identified clusters of non-vaccination in Moyamba and Bo districts. Overall, these factors suggested that the degree of general care provided to dogs, access to veterinary services, and the location of the dog owners were driving factors for vaccination.

We observed a vaccination rate of only 14% within the HHs. This was significantly below the WHO-recommended 70% threshold required to interrupt RABV transmission [31], and highlighted a significant challenge to ongoing disease control and canine rabies elimination. The vaccination rate obtained in this study was consistent with reports from Mali (17.6%) [32] and Chad (19%) [33]. However, it is lower compared to the rates observed in Nigeria (21%-70%) and Namibia 50% [34,35]. Potential factors contributing to low vaccination include low awareness of the importance of dog vaccination, the lack of veterinary establishments, and insufficient on-the-ground professionals in the country [13].

This highlights the role of education in enhancing the overall understanding of rabies and subsequently reducing the risk of RABV transmission. However, no significant difference was found when comparing the estimated probability of vaccination between individuals with tertiary education and those without formal education. Despite higher education impacting latent understanding, this suggests that access to vaccination remains a key issue in Sierra Leone. Indeed, the primary reason for non-vaccination among 2,219 respondents with unvaccinated dogs was a lack of knowledge on how to have their dogs vaccinated’ against rabies (70%, 1,574/2,219). In Namibia, where there has been huge investment in rabies prevention and control, a recent study found that 43.6% of dog-owning HHs with unvaccinated dogs reported not knowing how to vaccinate their dogs against rabies [35].

Furthermore, our result indicated that HHs in areas with a veterinary establishment were more likely to vaccinate their dogs against rabies than those without one. Similar finding was reported in Nigeria [36]. In addition to increasing awareness about rabies and the importance of vaccination, veterinary service facilities play an important role in the delivery of vaccination. Some identified challenges in developing countries, including Sierra Leone, are inadequate veterinary establishments and the limited number of practising veterinarians, who often work with inadequate equipment compared to those in developed nations [13,37]. Moreover, even where such facilities exist, they are often inadequately equipped compared to their counterparts in well-resourced countries [37,38]. This resource disparity hampers the delivery of essential veterinary services and exacerbates the struggle against diseases like rabies. Other factors include poor motivation of community and animal health workers [39].

Empowering community animal health workers through comprehensive training, community engagement, and mobile outreach programs can significantly boost rabies vaccination coverage and enhance veterinary capacity in areas with limited access to veterinarians [40].

In this study, occupations such as civil servants had a higher probability of dog vaccination than private workers and artisans, consistent with a recent report in Japan [41]. Civil servants may be more educated than private workers or artisans. Consequently, they may recognise the importance of canine rabies vaccination more than private workers or artisans. This was consistent with the result from Nigeria, which found that dog owners who were civil servants were 4.8 times more likely to have adequate knowledge [42]. Moreover, civil servants’ involvement in designing and implementing public health policies may motivate them to prioritise vaccinating their dogs, aligning with their professional responsibilities in promoting community health.

Our results indicated that dog owners residing in urban areas were more likely to vaccinate their dogs than those in rural areas. This finding was consistent with reports from Nigeria, Haiti and Namibia, which observed that dogs in urban areas were more likely to be vaccinated against rabies [17,35,43]. It also aligned with prior reports indicating a predominant focus of vaccination campaigns in urban areas (e.g., Freetown), leaving rural regions underserved [13]. Our cluster analysis supported this observation, revealing low-risk (for low vaccination) in Freetown, the capital of Sierra Leone, and high-risk (for low vaccination) districts like Moyamba, situated in rural parts of the country. Several factors may explain this trend, including the concentration of better veterinary facilities in urban areas, exemplified by Freetown’s sole private veterinary clinic and most international rabies control efforts directed towards the capital city [13]. Deficiencies of remoteness, often with inadequate road networks, also hamper efforts in rural enclaves. While rabies remains endemic throughout the country, the limited resources available necessitate strategic prioritisation of intervention locations. Future vaccination campaigns could benefit from utilising this information as a guide for prioritising locations for intervention.

Many respondents indicated they would consult traditional healers in the event of a dog bite and were less likely to vaccinate their dogs against rabies. Other studies reported similar findings in the region and elsewhere [44–46]. However, this contrasts with the report from Nambia, where only 8% of respondents indicated they would adopt traditional treatment in the event of a dog bite [35]. The observed trend in Namibia may be attributed to ongoing investments in rabies prevention and control over the years, which differs from the situation in Sierra Leone [10]. This highlights the dog owners’ lack of awareness of PEP. Utilising traditional treatments after a dog bite could result in fatalities for the owners/victims [47]. Targeted educational campaigns are urgently needed to enhance awareness about PEP and promote responsible pet ownership practices. Moreover, considering PEP is not readily available across Sierra Leone and the region, implementing Integrated Bite Case Management, as recently recommended in their national strategy for rabies control, is crucial to ensure timely access to PEP [48]. Implementing interventions that integrate traditional and modern perspectives is essential for a comprehensive strategy to enhance human and canine health [49].

The HHs with more dogs and those allowing their dogs to roam were less likely to vaccinate against rabies, a trend also observed recently in Nigeria [36]. Various factors, such as security, herding, and breeding, contribute to the inclination to own multiple dogs in Africa [17,45,50]. Despite existing legislation prohibiting unrestrained roaming (Animal Welfare Act), Sierra Leone faces a significant challenge with a large population of free-roaming dogs, hindering rabies prevention and control efforts. Our model indicated a lower vaccination probability among free-roaming dogs than those in confinement. This finding was consistent with a report from Morocco [45]. Given the socioeconomic challenges in Sierra Leone, a country grappling with economic difficulties, owning multiple dogs can place an added financial strain on dog owners [51]. To address this, initiatives should promote responsible pet ownership, prioritise pet welfare, and consider financial constraints. Sierra Leone could implement strategies from its National Strategic Plan, including spaying/neutering, mass dog vaccination, and legislation enforcement.

Our approach to modelling dog owners’ overall understanding of rabies as a latent variable was novel and robust. It offered an advancement over previous studies on rabies that modelled rabies knowledge or understanding using observed data [52]. This is because knowledge is always latent and can never be fully measured [53]. Similar approaches have been used in diverse fields to target latent knowledge or ability questions [53,54]. Our results suggested that latent understanding (dog owners’ overall knowledge of the disease) was positively associated with dog vaccination and tertiary-level education, consistent with a recent report from Nigeria [36].

A few limitations are worth highlighting when interpreting findings of this study. First, due to the cross-sectional nature of the study design, causal inferences cannot be established. Second, self-reported responses could be subjected to recall and response bias. Third, social desirability might have led to over-reporting some responses, such as caring for dogs. Despite these limitations, this study provides the first study on vaccination status in Sierra Leone, and the findings can be used for future vaccination programs.

In conclusion, our study reveals a significant deficiency in dog rabies vaccination rates, a scarcity of veterinary services, and suboptimal dog management practices. Our study highlights a critical need for public outreach and education, improved vaccination rates, and increased accessibility to veterinary services to support rabies prevention and control. Without such improvements, it is highly unlikely that Sierra Leone will reach the global goal of eliminating human deaths from dog-mediated rabies by 2030.

### Recommendations

There is a need to invest in training community animal health workers and veterinarians to augment the workforce and improve the delivery of vaccinations and healthcare for dogs throughout the country. Targeted educational campaigns, particularly for those with lower education levels, need to emphasise the importance of free dog vaccination, enhance awareness, and promote proactive behaviours in rabies prevention and control. Addressing the disparity in pet vaccination coverage is necessary. Targeted educational campaigns should be implemented to raise awareness about the efficacy of modern veterinary care, encourage responsible pet ownership practices, and discourage reliance on traditional healers for rabies-related issues. Finally, dog owners need to be encouraged to keep only the number of dogs they can adequately care for within their means.

## Supporting information

S1 Fig Map of Sierra Leone. The map was created using ArcMap software (ESRI Inc., Redlands, CA, U.S.A.). The shapefile was retrieved from DIVA-GIS (https://www.diva-gis.org/).
(DOCX)

S1 File Simulation of the expected data-generating process. (DOCX)
