# Supplementary figures and images for: Insights into canine rabies vaccination Disparities in Sierra Leone: A cross-sectional household study
Source: PLoS Negl Trop Dis. 2024 Jul 19;18(7):e0012332. doi: 10.1371/journal.pntd.0012332 (PMC11290662; doi:10.1371/journal.pntd.0012332)

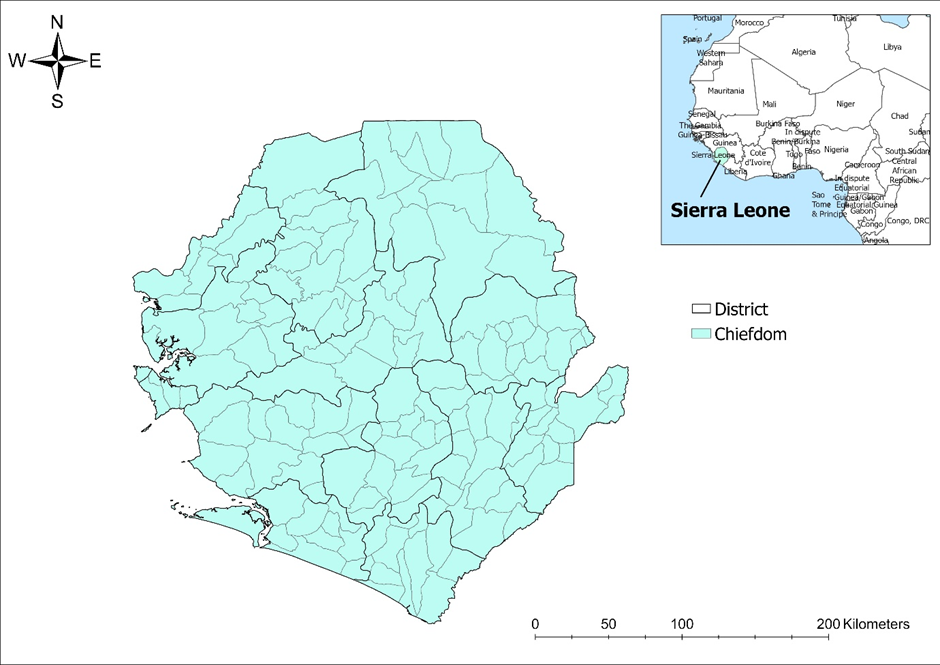


S1: Map of Sierra Leone

Supplement: S1 Fig — The map was created using ArcMap software (ESRI Inc., Redlands, CA, U.S.A.). The shapefile was retrieved from DIVA-GIS (https://www.diva-gis.org/). (DOCX) [file pntd.0012332.s001.docx]
